# Hypotensive and Vasorelaxant Effects of Sanguisorbae Radix Ethanol Extract in Spontaneously Hypertensive and Sprague Dawley Rats

**DOI:** 10.3390/nu15214510

**Published:** 2023-10-24

**Authors:** Jaesung Jung, Sujin Shin, Junkyu Park, Kyungjin Lee, Ho-Young Choi

**Affiliations:** 1 Department of Korean Medicine, Graduate School, Kyung Hee University, Seoul 02447, Republic of Korea; kingdom-js@daum.net (J.J.); sjshin04@khu.ac.kr (S.S.); 2 Department of Science in Korean Medicine, Graduate School, Kyung Hee University, Seoul 02447, Republic of Korea; ojeoksan@khu.ac.kr; 3 Department of Herbal Pharmacology, College of Korean Medicine, Kyung Hee University, Seoul 02447, Republic of Korea

**Keywords:** *Sanguisorba officinalis*, Sanguisorbae radix, vasorelaxant, NO/cGMP pathway, hypertension, angiotensin II, blood pressure

## Abstract

Hypertension requires proper management because of the increased risk of cardiovascular disease and death. For this purpose, functional foods containing tannins have been considered an effective treatment. Sanguisorbae radix (SR) also contains various tannins; however, there have been no studies on its vasorelaxant or antihypertensive effects. In this study, the vasorelaxant effect of the ethanol extract of SR (SRE) was investigated in the thoracic aorta of Sprague Dawley rats. SRE (1, 3, 10, 30, and 100 μg/mL) showed this effect in a dose-dependent manner, and its mechanisms were related to the NO/cGMP pathway and voltage-gated K^+^ channels. Concentrations of 300 and 1000 μg/mL blocked the influx of extracellular Ca^2+^ and inhibited vasoconstriction. Moreover, 100 μg/mL of SRE showed a relaxing effect on blood vessels constricted by angiotensin II. The hypotensive effect of SRE was investigated in spontaneously hypertensive rats (SHR) using the tail-cuff method. Blood pressure significantly decreased 4 and 8 h after 1000 mg/kg of SRE administration. Considering these hypotensive effects and the vasorelaxant mechanisms of SRE, our findings suggests that SRE can be used as a functional food to prevent and treat hypertension. Further studies are needed for identifying the active components and determining the optimal dosage.

## 1. Introduction

Hypertension has a worldwide prevalence of about 30% and requires management because of the increased risk of cardiovascular disease and death [1]. However, among patients receiving hypertension treatment, the rate of maintaining normal blood pressure is very low, at approximately 20% [2]. In addition, taking high blood pressure medications may cause side effects such as dizziness and palpitation [3] and increase the risk of hyperuricemia and gout [4]. Therefore, it is necessary to investigate alternative treatment methods to manage high blood pressure, including functional food and herbs [5,6,7,8].

*Sanguisorba officinalis* Linné is a plant belonging to the Rosaceae family and grows abundantly in Asia, Europe, and North America [9]. Young leaves and flower buds are eaten in salads, and the roots have long been used as herbs for the treatment of bleeding, internal hemorrhage, melena, burn, and dermatitis in the Republic of Korea, Japan, and China [10]. Sanguisorbae radix (SR), the root of *Sanguisorba officinalis*, has been used for its hemostatic effects [11], and many studies have described its anti-inflammatory [12], antioxidant [13], anti-cancer [14], and antidiabetic [15] effects.

The main components of SR are tannins [16], which have been primarily analyzed for their anticancer activity [17]. Studies have shown that tannins extracted from other plants act as vascular relaxants [18] and are effective in treating high blood pressure by inhibiting angiotensin-converting enzymes [19]. However, to the best of our knowledge, there are no studies on SR as a vascular relaxant or antihypertensive. 

Therefore, we investigated the vasorelaxant effect and its mechanism of the ethanol extract of SR (SRE) in the thoracic aortic ring of Sprague Dawley (SD) rats contracted with phenylephrine (PE) in this study. In addition, the hypotensive effects of SRE were investigated by measuring the blood pressure of spontaneously hypertensive rats (SHR) before and after SRE administration.

## 2. Materials and Methods

### 2.1. Materials and Chemicals

PE, acetylcholine (ACh), methylene blue (MB), indomethacin, ethyleneglycol-bis(2-aminoethylether)-N,N‚N′,N′-tetraacetic acid (EGTA), and angiotensin II (Ang II) were purchased from Sigma Aldrich (ST. Louis, MA, USA). 1H-[1,2,4]Oxadiazolo[4,3-a]quinoxalin-1-one (ODQ) was purchased from Tokyo Chemical Industry (Tokyo, Japan). NG-nitro-L-arginine methyl ester (L-NAME), 4-aminopyridine (4-AP), glibenclamide, and tetraethylammoniumchloride (TEA) were purchased from Wako Pure Chemical Industries (Osaka, Japan). Barium chloride (BaCl_2_), glucose, magnesium sulfate (MgSO_4_), monobasic potassium phosphate (KH_2_PO_4_), potassium chloride (KCl), sodium chloride (NaCl), sodium hydrogen carbonate (NaHCO_3_), calcium chloride (CaCl_2_), and urethane were purchased from Daejeong Chemical & Gold (Siheung-si, Republic of Korea). Dimethyl sulfoxide (DMSO) was purchased from Junsei (Tokyo, Japan), and ethanol was purchased from Duksan Pharmaceutical Co., Ltd. (Ansan-si, Republic of Korea).

### 2.2. Sample Preparation

*Sanguisorba officinalis* L. was collected in Pyeongchang-gun, Gangwon-do, Republic of Korea, in October 2021. Subsequent to the collection, morphological identification was performed by Professor Weon-Ki Paik, affiliated with the division of Life Science & Chemistry at Daejin University, and by Kang-Hyup Lee from the division of Forest Biodiversity at the Korea National Arboretum. To ascertain genetic identification, a comprehensive comparison of ITS, matK, and rbcL DNA barcodes was undertaken. The voucher specimen of *Sanguisorba officinalis* L. was deposited at the College of Korean Medicine, Kyung Hee University, Seoul, Republic of Korea. The root was naturally dried in a well-ventilated and shaded environment. A 10-fold amount (300 mL) of 50% ethanol was added to the dried SR (30 g), and extraction was performed by boiling at a consistent temperature of 70 ± 2 °C for 2 h. SRE was filtered twice with a qualitative filter paper (Hyundai micro, No. 2), freeze-dried, and stored in a −20 °C refrigerator. The extract yield was 23.3%. SRE was dissolved in DMSO and used in the experiments.

### 2.3. Animals

Male SD rats, weighing between 230 and 250 g and aged between 6 and 7 weeks (sourced from Daehan Biolink, Eumseong-gun, Republic of Korea), along with male SHR, weighing between 450 and 500 g and aged 1.5 years (sourced from SLC, Inc., Shizuoka, Japan), were employed in the study. The SHRs were randomly allocated to either the control group or the SRE administration group. The animals utilized in the experiments were accommodated in an environment maintained at a room temperature of 22 ± 2 °C, subjected to a 12/12 h light/dark cycle. Both feed and tap water were provided ad libitum. All experiments complied with the Animal Welfare Guidelines of the Animal Experiment Ethics Committee of Kyung Hee University and were approved by the committee (KHSASP-23-066).

### 2.4. Measurement of Isotonic Changes in Blood Vessels

#### 2.4.1. Preparation of Rat Aortic Rings

SD rats were anesthetized by intraperitoneal injection of urethane (1.2 g/kg), and the thoracic aorta was isolated. The isolated aorta was transferred to a Petri dish containing Krebs–Henseleit (KH) buffer composed of 118.0 mM NaCl, 4.7 mM KCl, 2.5 mM CaCl_2_, 1.2 mM MgSO_4_, 1.2 mM KH_2_PO_4_, and 25.0 mM NaHCO_3_. The connective tissue and fat surrounding the aorta were removed immediately, and it was cut into several 2–3 mm long segments. The cut aortic rings were hung between tungsten hooks in an organ bath containing 10 mL of KH buffer. A mixed gas of 95% O_2_ and 5% CO_2_ was continuously supplied and the organ bath was maintained at 37 °C. Changes in the isotonic contraction of blood vessels were recorded using PowerLab (AD Instrument Co., Bella Vista, Australia). The rings were stabilized in the organ bath for 40 min and were loaded with a passive tension of 1.0 g. During the equilibration period, the KH buffer in the organ bath was replaced with fresh KH buffer every 5–10 min.

#### 2.4.2. Vasodilatory Effect of SRE on the Rat Aortic Rings Constricted by PE

Thoracic aortic rings were pre-constricted with PE (1 μM) after the equilibration period to evaluate the vasodilatory effect of SRE. After reaching the maximal constriction and achieving equilibrium, SRE was added to the organ bath at cumulative concentrations (1, 3, 10, 30, and 100 μg/mL).

#### 2.4.3. Vasodilatory Effect of SRE on Endothelium-Intact and -Removed Aortic Rings

Thoracic aortic rings were pre-constricted with PE (1 μM) and then were relaxed with ACh (10 μM) to identify vascular endothelial cells. More than 85% relaxation by ACh was confirmed to have no endothelial damage, and aortic rings with less than 10% relaxation were confirmed to have no endothelium. After confirming the integrity of endothelial cells, the thoracic aortic rings were washed with KH buffer several times and constricted again with PE (1 μM).

#### 2.4.4. Vasodilatory Effect of SRE When Pretreated with L-NAME and Indomethacin

Following pretreatment with L-NAME (NO synthase inhibitor, 100 μM) and indomethacin (COX inhibitor, 10 μM) for 20 min, thoracic aortic rings were contracted with PE (1 μM), and then, SRE was administered at cumulative concentrations (1, 3, 10, 30, and 100 μg/mL).

#### 2.4.5. Vasodilatory Effect of SRE When Pretreated with ODQ and MB

After being pretreated with ODQ (sGC inhibitor, 10 μM) and MB (cGMP inhibitor, 10 μM) for 20 min, the aortic vessels were contracted with PE (1 μM), and then, SRE was administered at cumulative concentrations (1, 3, 10, 30, and 100 μg/mL).

#### 2.4.6. Vasodilatory Effect of SRE When Pretreated with K^+^ Channel Blockers

Pretreatment of vessels with BaCl_2_ (inward rectifier K^+^ channel blocker, 10 μM), 4-AP (voltage-dependent K^+^ channel blocker, 1 mM), TEA (Ca^2+^-dependent K^+^ channel blocker, 1 mM), and glibenclamide (ATP-dependent K^+^ channel blocker, 10 μM) was carried out for 20 min. After pretreatment, thoracic aortic rings were contracted with PE (1 μM), and then SRE was administered at cumulative concentrations (1, 3, 10, 30, and 100 μg/mL).

#### 2.4.7. Inhibitory Effect of SRE on Extracellular Ca^2+^-Induced Contraction

The aortic rings were stabilized in Ca^2+^-free KH buffer containing EGTA (1 mM) and were pre-incubated with SRE (100, 300 and 1000 μg/mL) for 20 min. Following PE (1 μM) treatment for 20 min, CaCl_2_ (0.1, 0.3, 1, 3, 10 mM) was sequentially introduced. The inhibitory effect of SRE on vasoconstriction induced by Ca^2+^ channels was compared to that of the control group without SRE.

#### 2.4.8. Inhibitory Effect of SRE on Ang II-Induced Contraction

The aortic rings were stabilized in KH buffer and then pre-incubated with SRE (100 μg/mL) for 20 min. Ang II was administered at cumulative concentrations (10^−9^–10^−6^ M), and the inhibitory effect of SRE on vasoconstriction induced by Ang II was compared to that of the control group without SRE.

### 2.5. Blood Pressure Measurement of SHR

The systolic blood pressure (SBP) and diastolic blood pressure (DBP) of the SHR were measured using the tail-cuff method (CODA 8-Channel High Throughput Non-Invasive Blood Pressure System, Kent Scientific Co., Torrington, CT, USA). SRE (300 mg/kg and 1000 mg/kg per dose) was administered orally to the SHR. SBP and DBP were measured before and 1, 2, 4, and 8 h after drug administration and then compared with the control group (administered distilled water instead of SRE). 

### 2.6. Statistical Analysis

All statistical analyses were performed using an unpaired *t*-test and two-way ANOVA using GraphPad Prism 9 software (San Diego, CA, USA). Bonferroni’s multiple comparison test was used for post hoc analysis. The experimental data were presented as the mean ± standard error of the mean (SEM). Statistical significance was confirmed at *p* < 0.05.

## 3. Results

### 3.1. Vasodilatory Effect of SRE on the Aortic Rings Constricted by PE

**Figure 1:**
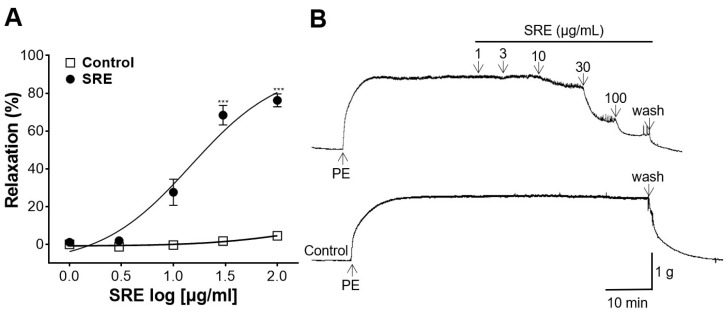
Vasorelaxant effect of Sanguisorbae radix 50% ethanol extract (SRE) on rat thoracic aortic rings pre-constricted with phenylephrine (PE, 1 μM). (**A**) Cumulative concentration–response curves and (**B**) representative traces of rat aortic rings. Values are expressed as mean ± SEM (*n* = 4–5). *** *p* < 0.001 vs. control.

Aortic rings were constricted with PE (1 μM) and treated with SRE (1, 3, 10, 30, and 100 μg/mL). The resulting vasorelaxant effect occurred in a concentration-dependent manner compared to the control (Figure 1). SRE at 100 μg/mL showed a maximum effect of 74.81 ± 3.00% on PE-constricted aortic rings.

### 3.2. Vasodilatory Effect of SRE on Endothelium-Intact and -Removed Aortic Rings

**Figure 2:**
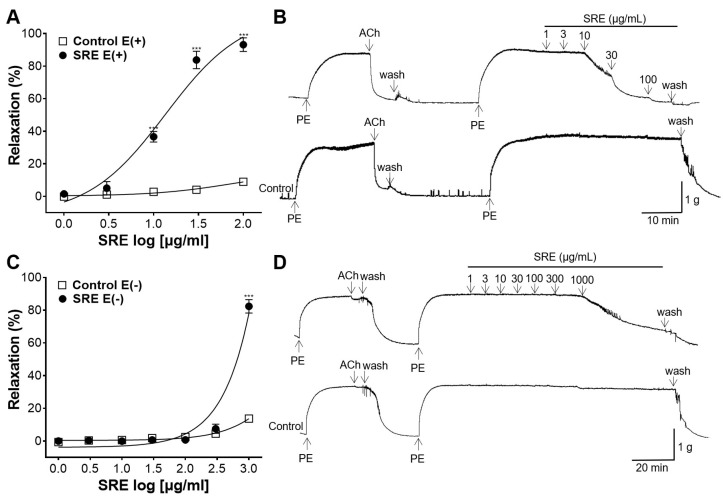
Vasorelaxant effect of Sanguisorbae radix 50% ethanol extract (SRE) on endothelium-intact [E(+)] or endothelium-removed [E(−)] rat thoracic aortic rings pre-constricted with phenylephrine (PE, 1 μM). (**A**,**C**) Cumulative concentration-response curves and (**B**,**D**) representative traces of rat aortic rings. Values are expressed as mean ± SEM (*n* = 4). *** *p* < 0.001 vs. control.

Endothelium-intact and -removed aortic rings were compared with each control group to confirm whether the vasorelaxant effect of SRE was related to endothelial cells. In the presence of the endothelium cells, SRE acted as a vasorelaxant in a concentration-dependent manner, with a maximum effect of 93.18 ± 4.20% at 100 μg/mL (Figure 2A,B). However, in the absence of cells, no significant changes were observed until the dose reached 300 μg/mL. Maximum relaxation was observed after treatment with 1000 μg/mL of SRE (Figure 2C,D).

### 3.3. Effects of L-NAME and Indomethacin Pretreatment on Vasorelaxation

**Figure 3:**
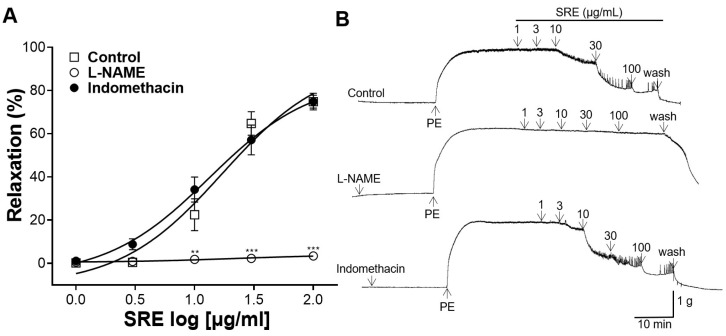
Vasorelaxant effect of Sanguisorbae radix 50% ethanol extract (SRE) on rat thoracic aortic rings pre-incubated with N^G^-nitro-L-arginine methyl ester (L-NAME, 100 μM) and indomethacin (10 μM). (**A**) Cumulative concentration–response curves and (**B**) representative traces of rat aortic rings. Values are expressed as mean ± SEM (*n* = 4–5). ** *p* < 0.01, *** *p* < 0.001 vs. control.

Thoracic aortic rings were pretreated with L-NAME (100 μM) and indomethacin (10 μM), and the vasorelaxant effect of SRE (1, 3, 10, 30, and 100 μg/mL) was compared with that of the control (without pretreatment) group. Pretreatment with L-NAME significantly suppressed the vasorelaxant effect of SRE (Figure 3). However, pretreatment with indomethacin showed no significant difference with the control group (Figure 3).

### 3.4. Effects of ODQ and MB Pretreatment on Vasorelaxation

**Figure 4:**
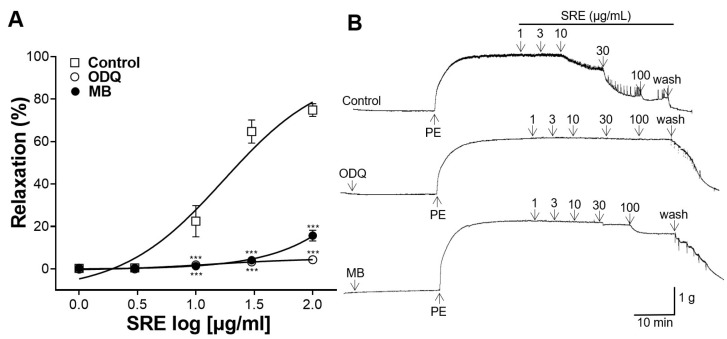
Vasorelaxant effect of Sanguisorbae radix 50% ethanol extract (SRE) on rat thoracic aortic rings pre-incubated with 1H-[1,2,4]Oxadiazolo[4,3-a]quinoxalin-1-one (ODQ, 10 μM) and methylene blue (MB, 10 μM). (**A**) Cumulative concentration–response curves and (**B**) representative traces of aortic rings. Values are expressed as mean ± SEM (*n* = 4–5). *** *p* < 0.001 vs. control.

Thoracic aortic rings were pretreated with ODQ (10 μM) and MB (10 μM), and the vasorelaxant effect of SRE (1, 3, 10, 30, and 100 μg/mL) was compared with that of the control (without pretreatment) group. The vasodilatory effect was significantly suppressed in aortic rings pretreated with ODQ and MB compared to the control group (Figure 4).

### 3.5. Effect of K^+^ Channel Blocker Pretreatment on Vasorelaxation

**Figure 5:**
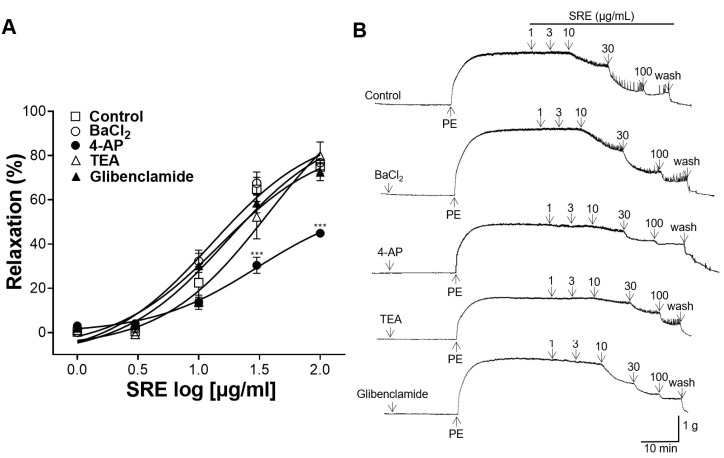
Vasorelaxant effect of Sanguisorbae radix 50% ethanol extract (SRE) on rat thoracic aortic rings pre-incubated with barium chloride (BaCl_2_), 4-aminopyridine (4-AP), tetraethylammonium (TEA), or glibenclamide. (**A**) Cumulative concentration–response curves and (**B**) representative traces of rat aortic rings. Values are expressed as mean ± SEM (*n* = 4–7). *** *p* < 0.001 vs. control.

Thoracic aortic rings were pretreated with K^+^ channel blockers: BaCl_2_ (10 μM), 4-AP (1 mM), TEA (1 mM), and glibenclamide (10 μM). The vasorelaxant effect of SRE (1, 3, 10, 30, and 100 μg/mL) was compared with that of the control (without pretreatment) group. The group pretreated with BaCl_2_, glibenclamide, and TEA showed no significant differences compared to the control group, but the vasorelaxant effect of SRE was significantly suppressed by pretreatment with 4-AP (Figure 5).

### 3.6. Inhibitory Effect of SRE on Extracellular Ca^2+^-Induced Contraction

**Figure 6:**
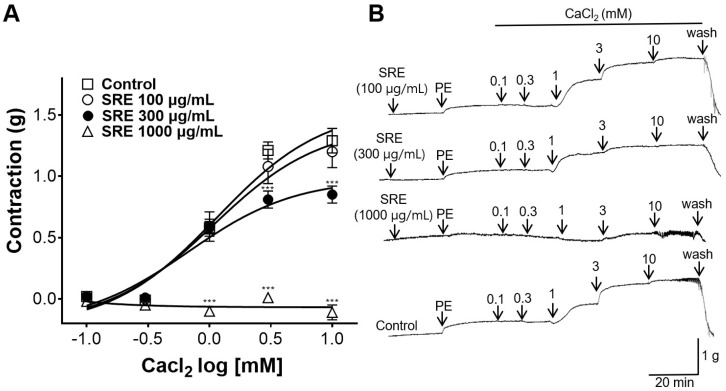
Inhibitory effect of Sanguisorbae radix 50% ethanol extract (SRE) on rat thoracic aortic rings constricted by extracellular Ca^2+^. (**A**) Effect on extracellular CaCl_2_ (0.1, 0.3, 1, 3, and 10 mM) constriction and (**B**) representative traces of aortic rings. Values are expressed as mean ± SEM (*n* = 4). *** *p* < 0.001 vs. control.

Thoracic aortic rings were pretreated with SRE (100, 300, and 1000 μg/mL), and CaCl_2_ (0.1, 0.3, 1, 3, and 10 mM) was administered for gradual contraction. No significant differences were observed between the group treated with 100 µg/mL of SRE and the non-treated control group (Figure 6). However, 300 and 1000 µg/mL of SRE showed inhibitory effect on Ca^2+^-induced contraction compared to the non-treated control group.

### 3.7. Inhibitory Effect of SRE on Angiotensin II-Induced Contraction

**Figure 7:**
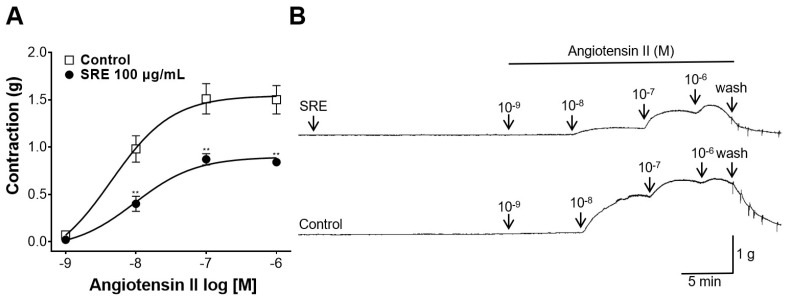
Inhibitory effect of Sanguisorbae radix 50% ethanol extract (SRE) on rat thoracic aortic rings constricted by angiotensin II (Ang II). (**A**) Inhibitory effect on Ang II (10^−9^–10^−6^ M) constriction and (**B**) representative traces of aortic rings. Values are expressed as mean ± SEM (*n* = 5). ** *p* < 0.01 vs. Control.

Thoracic aortic rings were pretreated with 100 µg/mL of SRE, and Ang II was administered at cumulative concentrations (10^−9^, 10^−8^, 10^−7^, and 10^−6^ M). Consequently, 100 µg/mL of SRE inhibited Ang II-induced contractions compared to the control group (Figure 7).

### 3.8. Blood Pressure-Lowering Effect of SRE

**Figure 8:**
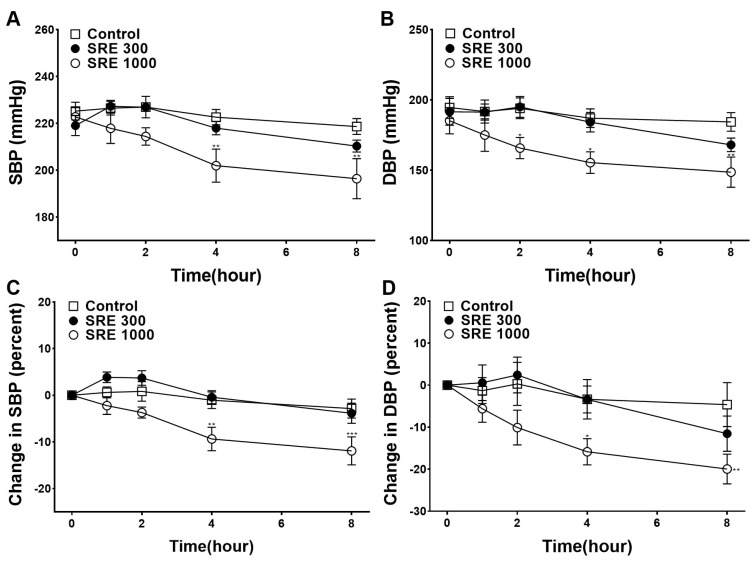
Changes in blood pressure by Sanguisorbae radix 50% ethanol extract (SRE) administration in spontaneously hypertensive rats (SHR). (**A**) Systolic blood pressure (SBP), (**B**) diastolic blood pressure (DBP), (**C**) percent changes in SBP, (**D**) percent changes in DBP. Values are expressed as mean ± SEM (*n* = 5). * *p* < 0.05, ** *p* < 0.01, *** *p* < 0.001 vs. control.

To investigate the blood pressure-lowering effect of SRE, SBP and DBP were measured at 1, 2, 4, and 8 h after the oral administration of 300 or 1000 mg/kg of SRE to SHR. The measured SBP and DBP values were compared with the control group. Because of pre-existing differences in blood pressure in individual SHRs, the difference before and after administration was calculated and was compared with the control group. The SBP and DBP values significantly decreased 4 and 8 h after the administration of 1000 mg/kg of SRE (Figure 8).

## 4. Discussion

In the present study, we evaluated the vasorelaxant and hypotensive effects of SRE. The vasorelaxant effect and its mechanism of action were investigated using dissected thoracic aorta of SD rats constricted with PE. Our results showed that SRE relaxed the PE-constricted thoracic aortic rings in a concentration-dependent manner compared to the control group. Various factors affect vasodilation. Among them, vascular endothelial cells play a crucial role in regulating vascular relaxation and contraction, thereby maintaining circulatory homeostasis [20]. To determine whether endothelial cells are involved in the vascular relaxation of SRE, the vasodilatory effect was evaluated by administering SRE on thoracic aortic rings with and without endothelial cells. The experimental results showed that SRE (1, 3, 10, 30, and 100 μg/mL) relaxed PE-constricted thoracic aortic rings with endothelium in a concentration-dependent manner. However, in the experiment in which the endothelium was removed, no significant effect was seen at concentrations below 300 µg/mL, whereas vascular relaxation was observed at a concentration of 1000 µg/mL. These results indicate that SRE has an endothelium-dependent vasorelaxant effect at low concentrations (below 300 µg/mL) and has an additional vasorelaxant effect through an endothelium unrelated mechanism at higher concentrations.

NO and PGI_2_ are released from endothelial cells and cause VSMC relaxation [21]. NO is produced from L-arginine by NO synthase, and PGI_2_ is produced from arachidonic acid by COX [21]. To study the mechanism of action of SRE in vascular endothelial cells, thoracic aortic sections were pretreated with L-NAME (an NO synthase inhibitor) and indomethacin (a COX inhibitor), and contraction was induced with PE. In our study, the vasorelaxant effect was significantly reduced in the aortic rings pretreated with L-NAME; however, there was no significant difference compared to the control in the aortic rings with indomethacin pretreatment. Therefore, our results indicate that the vasorelaxant effect of SRE is not related to PGI_2_ but is related to NO.

The NO produced in vascular endothelial cells activates sGC in VSMC and increases cGMP concentration [22]; consequently, Ca^2+^ in VSMC decreases, causing blood vessels to relax [21]. To determine whether SRE acts on the NO/cGMP pathway, the thoracic aortic rings were pretreated with ODQ (an sGC inhibitor) or MB (a cGMP inhibitor). In our study, pretreatment with ODQ and MB significantly reduced the vasorelaxant effect, indicating that SRE relaxes blood vessels through the NO/cGMP pathway in vascular endothelial cells.

K^+^ channels are important regulators of VSMC [23]. When K^+^ channels are activated, K^+^ efflux increases and the activity of voltage-dependent Ca^2+^ channels is reduced, thereby reducing the intracellular Ca^2+^ concentration [24]. To study the mechanism of action of SRE on K^+^ channels, thoracic aortic sections were pretreated with BaCl_2_ (an inward rectifier K^+^ channel blocker), 4-AP (a voltage-dependent K^+^ channel blocker), TEA (a Ca^2+^-dependent K^+^ channel blocker), or glibenclamide (an ATP-dependent K^+^ channel blocker), and the vasorelaxant effects were compared to those in the control group. The BaCl_2_, TEA, and glibenclamide pretreatments did not affect the vasorelaxant effect of SRE; however, 4-AP pretreatment significantly inhibited it. These results indicated that the vasorelaxant effect of SRE is related to voltage-gated K^+^ channels (K_v_).

Ca^2+^ channels are also important mechanisms that regulate the contraction and relaxation of VSMC. When intracellular Ca^2+^ concentration increases, actin–myosin interactions increase, leading to VSMC constriction [25]. To investigate whether SRE activity is related to Ca^2+^ channels, the thoracic aortic rings were pretreated with SRE in a Ca^2+^-free KH buffer. The thoracic aortic rings were then constricted with PE, and CaCl_2_ was administered cumulatively (0.1, 0.3, 1, 3, and 10 mM). In our experiment, there was no significant difference between the 100 µg/mL SRE group and the non-treated control group. However, SRE concentrations of 300 and 1000 µg/mL showed significant difference from the control group. This suggests that SRE inhibits constriction via extracellular Ca^2+^ influx at high doses (300 and 1000 µg/mL).

Furthermore, Ang II, which is produced in the vascular wall, is also involved in regulating vascular tone [26]. Ang II induces vasoconstriction by increasing intracellular Ca^2+^ concentrations, and the dysregulation of Ang II contributes to the development of hypertension [27]. To investigate whether SRE activity affects Ang II, the thoracic aortic rings were pretreated with 100 µg/mL of SRE and Ang II was administered cumulatively (10^−9^–10^−6^ M). Consequently, SRE significantly reduced the degree of contractility induced by Ang II, proving its inhibitory effect on the peptide.

To determine whether SRE is effective in treating high blood pressure in vivo, SBP and DBP were measured 1, 2, 4, and 8 h after oral administration of SRE (300 or 1000 mg/kg) in SHR. After 4 and 8 h of SRE 1000 mg/kg of SRE, SBP and DBP significantly decreased. When converting the dose administered to animals (300 mg/kg–1000 mg/kg) to the human equivalent, approximately 3–10 g of SRE can be added to an adult male weighing 60 kg [28]. SRE has long been used in traditional medicine for its hemostatic effects [11]. In general, natural products used in traditional medicine in east Asia showed a wide range of therapeutic dosage (e.g., 4–80 g) and toxicities were known to be low [29]. However, further investigation and toxicity evaluation are required to determine the optimal administration dosage.

The prevalence of high blood pressure increases with age and is accompanied by endothelial cell damage [30]. Therefore, it is important to determine whether it lowers blood pressure in older adults. In our study, the SHR were older (1.5 years old), and it was assumed that their vascular endothelial cells were impaired [31]. Because SRE has a vasorelaxant effect through various mechanisms as well as an endothelium-related relaxation mechanism, it appears to be effective in lowering blood pressure in older SHR. Considering these results, SRE could be used for the prevention and treatment of high blood pressure.

According to a previous study, SRE has bioactive compounds belonging to tannins, flavonoids, and phenolic acids [32]. In particular, SRE is widely known to be rich in tannins, primarily hydrolysable ellagitannins and gallotanins, such as sanguiin H-1, sanguiin H-2, sanguiin H-6, and sanguiin H-10 [33]. Tannins extracted from other plants showed vasorelaxant and hypotensive effects; the tannin components of SRE are presumed to have the chief vasodilatory effect [18,19]. However, bioactive compounds in SRE were not analyzed in this study. Additional research is needed to identify the representative components in SRE that exhibit vasorelaxant and blood pressure-lowering effects.

As high blood pressure becomes increasingly prevalent, research is being continued to investigate methods for blood pressure management through food or plants. Studies showed that the vasodilatory effect of plant and natural products were mainly correlated with the NO/cGMP pathway or blockage of the Ca^2+^ channel [34]. Some natural products, such as anthocyanin-rich sour cherries [6] and *Prunus Persica* branches [35] showed endothelial-dependent vasorelaxation via the NO/cGMP pathway; other compounds, such as the persimmon-derived polyphenol phytocomplex [36] and scutellarin extracted from *Erigeron breviscapus* [37] showed vasorelaxation via the Ca^2+^ channel. Therefore, SRE appears to have an advantage over other foods or plants because it exhibits vascular relaxant effect via various mechanisms.

## 5. Conclusions

In conclusion, SRE activated the NO/cGMP pathway and blocked voltage-gated K^+^ channels (K_v_) to relax constricted blood vessels. At high concentrations, SRE blocked the influx of extracellular Ca^2+^ and inhibited blood vessel constriction. Moreover, SRE had a relaxing effect on blood vessels constricted by Ang II. The SBP and DBP of elderly SHR significantly decreased 4 and 8 h after administering 1000 mg/kg of SRE. Considering these vascular relaxation mechanisms and the hypotensive effect of SRE, our findings suggest that SRE can be used as a functional food to prevent and treat hypertension. However, more extensive research is imperative for further applications of SRE, including identifying its constituents and active components, determining the optimal dosage for administration, and evaluating its toxicity.

## Data Availability

The data presented in this study are available from the corresponding author upon request.
